# Cholestatic Hepatitis: A Rare Manifestation of Infectious Mononucleosis

**DOI:** 10.7759/cureus.72925

**Published:** 2024-11-03

**Authors:** Francisco Barreto, Sofia Nóbrega, Carolina Carvalhinha, Carolina Henriques, Teresa Faria

**Affiliations:** 1 Internal Medicine, Hospital Central do Funchal, Funchal, PRT

**Keywords:** cholestasis, cholestatic hepatitis, epstein-barr virus, hepatitis, jaundice

## Abstract

One of the main clinical manifestations of infection by the Epstein-Barr virus (EBV) is infectious mononucleosis. In this clinical syndrome, mild hepatitis with a slight elevation of aminotransferases is common. However, cholestasis is rare and usually occurs alongside a more severe, cytolytic hepatitis.

This case report describes a 26-year-old male admitted to the emergency service with recurrent fever, odynophagia, and painful cervical lymphadenopathy, along with a skin rash after starting treatment with amoxicillin/clavulanate, and jaundice. The analytical assessment was consistent with cholestatic hepatitis, and the abdominal CT scan revealed hepatosplenomegaly. The diagnosis of EBV infection was confirmed by the presence of serological markers. This case aims to raise awareness of a rare manifestation of a common infectious agent in order to consider acute EBV infection in the differential diagnosis of cholestatic hepatitis in adult patients.

## Introduction

The Epstein-Barr virus (EBV), or human herpesvirus 4 (HHV4), affects about 90% of the global population and is commonly associated with infectious mononucleosis (IM), which typically presents with a triad of fever, pharyngitis, and lymphadenopathy, and has a usually self-limited course [1]. Transmission occurs through oropharyngeal secretions [2]. In adults, primary infection can manifest in more severe and varied forms [3]. Generally, it initially occurs with asthenia, anorexia, and myalgias. Splenomegaly and/or hepatomegaly may be observed [3,4]. The diagnosis of IM is presumptive, based upon the association of typical symptoms and the presence of atypical lymphocytes in the peripheral blood [2-4]. It can be confirmed by serological tests that are divided into nonspecific tests, such as heterophile antibodies, and specific tests, which include antibodies against EBV antigens [4].

Although a subclinical and transient increase in hepatic aminotransferases is relatively common, cases of cholestatic hepatitis with marked jaundice are rare, with an overall incidence of less than 5% [5].

## Case presentation

**Figure 1:**
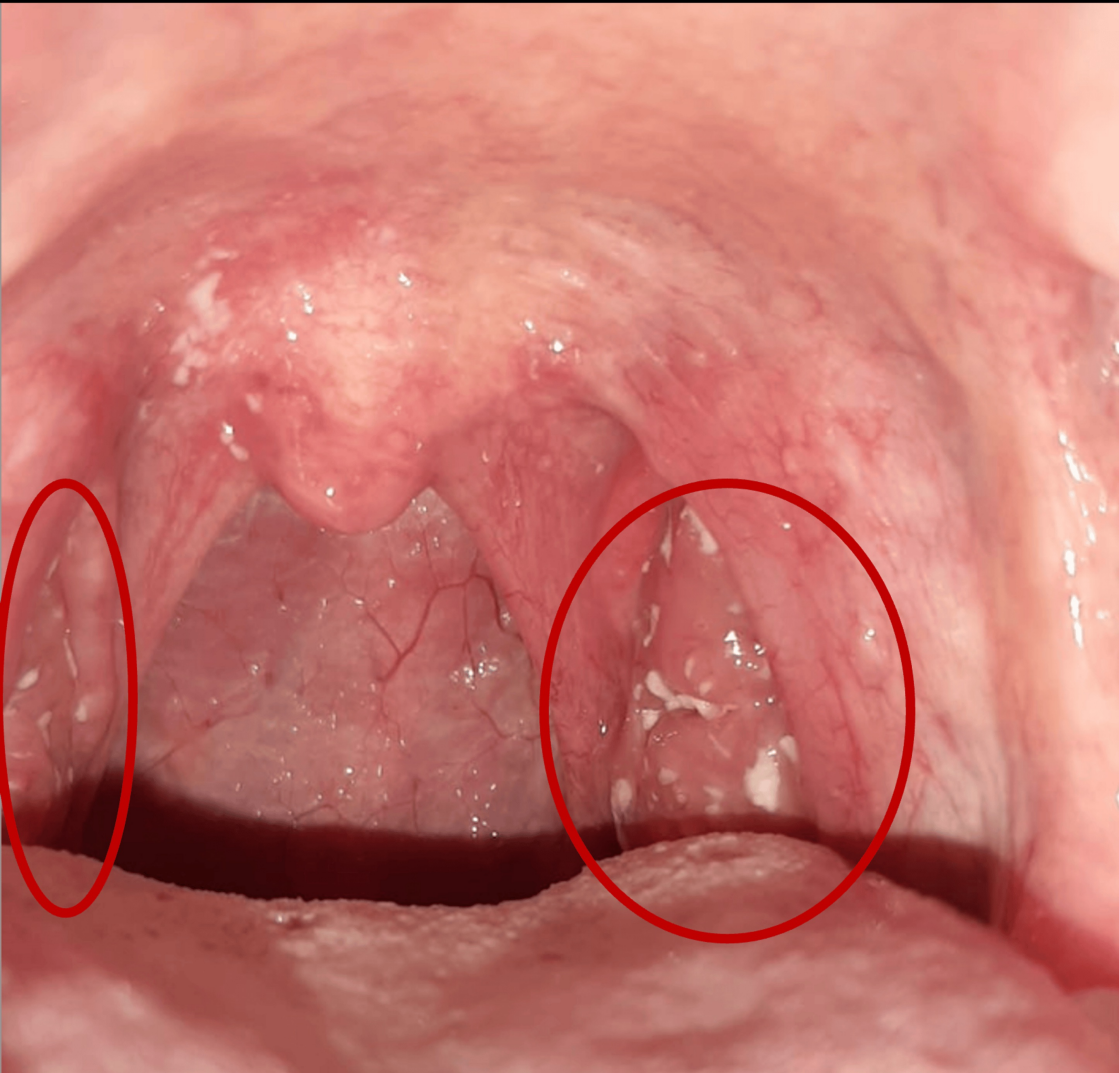
Pharyngotonsillitis Red circles indicate increased redness and an exudate covering the tonsil.

A 26-year-old male, with no significant personal medical history, went to the Emergency Department with a one-week history of odynophagia, recurrent fever, and painful cervical lymphadenopathy. He had already been treated with amoxicillin/clavulanate 875/125 mg orally and ibuprofen 400 mg every eight hours for the last two days due to suspicion of bacterial tonsillitis. The patient mentioned no improvement and had subsequently developed a pronounced non-itchy rash at the base of the neck and chest, along with diffuse abdominal pain. The physical examination revealed icteric sclerae and skin, tonsillar hypertrophy with purulent tonsillar exudate (Figure 1), painful cervical lymphadenopathy, erythema at the base of the neck and chest, pulmonary auscultation showing decreased vesicular breath sounds at the right lung base, and painful hepatosplenomegaly upon abdominal palpation.

**Figure 2:**
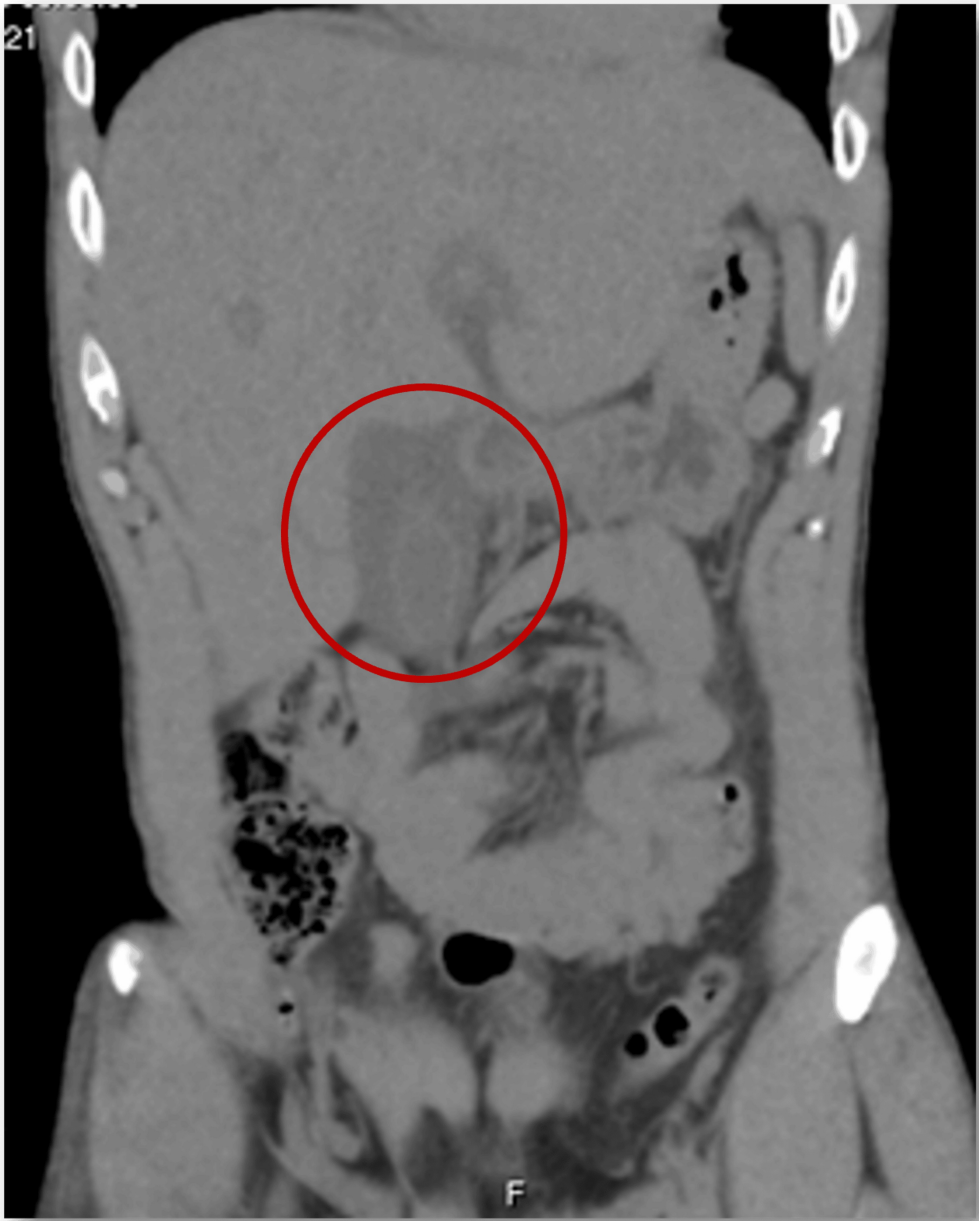
Homogeneous hepatomegaly The red circle demonstrates a moderately distended gallbladder with an exuberant edematous wall thickeness.

As shown in Tables 1-3, blood tests presented with hemoglobin of 13.2 g/dL, leukocytes of 6,600/μL, and 25% activated lymphocytes detected by flow cytometry analysis; thrombocytopenia of 102,000; hypoalbuminemia of 31.3 g/L; prothrombin time of 14.5 seconds; hepatic cytolysis with increased aspartate aminotransferase (AST) of 818.7 U/L, and alanine aminotransferase (ALT) of 966.9 U/L; mixed hyperbilirubinemia with total bilirubin of 6.85 mg/dL, direct bilirubin of 3.86 mg/dL, and indirect bilirubin of 2.99 mg/dL; lactate dehydrogenase (LDH) of 904 U/L; alkaline phosphatase (ALP) of 755.0 U/L; gamma-glutamyl transferase (GGT) of 648.3 U/L; and C-reactive protein (CRP) of 35 mg/dL. The urine dipstick test did not show bile pigments. The abdominal computed tomography with contrast revealed pronounced homogeneous hepatomegaly with periportal edema, a moderately distended gallbladder without endoluminal lithiasis, and exuberant edematous wall thickening (Figure 2).

**Table 1:** Hemogram dL: Deciliter; g: Gram; µL: Microliter

Hemogram	Results	Normal range
Hemoglobin (g/dL)	13.2	13.5-16.5
Leukocytes (/mm^3^)	6,600	3,500-10,000
Atypical lymphocytes (%)	25	<10
Platelets (/µL)	102,000	150,000-400,000

**Table 2:** Coagulation tests INR: International normalized ratio

Coagulation tests	Results	Normal range
Prothrombin time (seconds)	14.5	11-14
INR	1.2	0.8-1

**Table 3:** Biochemistry ALT: Alanine aminotransferase; ALP: Alkaline phosphatase; AST: Aspartate aminotransferase; CRP: C-reactive protein; γGT: Gamma-glutamyl transferase; LDH: Lactate dehydrogenase; dL: Deciliter; g: Gram; mg: Milligram

Biochemistry	Results	Normal range
Albumin (g/L)	31.3	35-54
ALT (U/L)	966.9	29-33
AST (U/L)	818.7	5-40
LDH (U/L)	904	120-246
Total bilirubin (mg/dL)	6.85	0.2-1.2
Direct bilirubin (mg/dL)	3.86	<0.3
Indirect bilirubin (mg/dL)	2.99	0.2-0.8
ALP (U/L)	755	30-120
GGT (U/L)	648.3	9-48
CRP (mg/dL)	35	<5.0

Blood heterophile antibodies were detected. Within the EBV tests, the patient presented positive viral capsid antigen (VCA) IgM and IgG, and negative Epstein-Barr nuclear antigen (EBNA) and early antigen (EA). The remaining serologies showed negative results for human immunodeficiency virus (HIV), hepatitis B virus (HBV), hepatitis C virus (HCV), and syphilis, with positive results for IgG for herpes simplex virus type 1 (HSV-1) and IgG for cytomegalovirus (CMV).

The patient was admitted for supportive care (fluidotherapy, rest, and analgesia) and monitoring. There was a significant clinical and analytical improvement, and the patient was discharged in one week with preventive measures and work avoidance.

In the follow-up consultation, there was analytical evidence of seroconversion, with positive EBNA, negative IgM VCA, and positive IgG VCA at higher titers.

## Discussion

EBV infection is most frequently diagnosed in the pediatric population, with IM being the classic clinical manifestation [6]. The severity of the symptoms is related to the immune response; therefore, it is often a subclinical disease during childhood and more severe in adolescents and adults [6,7].

Jaundice is a rare manifestation of EBV infection and is more common in people older than 35 years. It can be due to autoimmune hemolytic anemia or cholestasis from acalculous cholecystitis, biliary duct obstruction due to abdominal lymphadenopathy, or cholestatic hepatitis [8].

In this case, the patient presented with typical IM, a related skin rash following treatment with amoxicillin, and jaundice accompanied by hyperbilirubinemia in the absence of anemia. Hence, hemolytic anemia seems to be an unlikely cause of this clinical condition. An abdominopelvic CT scan has also ruled out extrahepatic cholestasis.

EBV infection is usually associated with mild and self-limited hepatitis, but there are reports of incidence as high as 55% of severe cholestatic hepatitis in adults [7]. Severe liver injury, although rare, is the leading cause of death in cases of acute EBV infection [7].

In the present case, the predominantly direct hyperbilirubinemia (82%), associated with significant elevations of γ-GT, AST, and ALT, indicates mixed liver injury, consistent with cholestatic hepatitis, which typically presents with aminotransferase values up to seven times above the upper limit of normal [9]. The liver cell damage mechanism that leads to cholestasis has not been clarified yet, given that EBV does not cause direct cytotoxic effects on hepatocytes. Cholestasis is possibly related to lipid peroxidation, with a consequent increase in free radicals, which can explain our patient's clinical presentation [10,11]. Drug-induced liver injury (DILI) is also an entity that has an increasing incidence in the last decade and can manifest as cholestatic hepatitis. Amoxicillin/clavulanate seems to be one of the most common causative agents of DILI [12]. In this case, we could also speculate about the contribution of amoxicillin in a possible DILI, which could explain the patient's moderate to severe cholestasis.

The approach to patients with cholestatic hepatitis due to EBV is primarily symptomatic treatment, including analgesia and control of pruritus when necessary. The course of the disease is often self-limited, with spontaneous resolution of signs and symptoms. The use of corticosteroids and antivirals is usually reserved for severe cases, such as fulminant hepatitis; nonetheless, their use remains controversial [11]. Moreover, there is a lack of strong evidence for the use of medications such as acyclovir, ganciclovir, or famciclovir in the management of these patients [11].

Most cases of cholestatic hepatitis do not present the other typical symptoms of EBV infection, making early diagnosis difficult [13]. Our patient exhibited typical clinical signs of IM with associated jaundice, and serologies confirmed acute infection by EBV and subsequent confirmation of seroconversion. The patient showed spontaneous resolution of the signs and symptoms, as well as the laboratory markers, with the supportive treatment implemented.

## Conclusions

The authors report a rare clinical manifestation of EBV infection presenting as IM with cholestasis. Key clinical findings, such as odynophagia, fever, hepatomegaly, and rash, raised suspicion of EBV infection. Considering EBV in cholestatic hepatitis is crucial to avoid unnecessary diagnostics and treatments. Moreover, DILI is increasingly diagnosed, and IM patients often receive antibiotics like amoxicillin/clavulanate, as seen in this case. Evaluating the role of medications, especially antibiotics, is essential in assessing hepatic and cholestatic injuries.

IM, even when associated with cholestasis and hepatic cytolysis, is typically a self-limited condition that resolves with supportive care. Steroid therapy may be considered in the management of complications associated with EBV infection, particularly in cases of fulminant hepatitis or septic shock.
